# Developing and Evaluating a Quality Improvement Intervention to Facilitate Patient Navigation in the Accountable Health Communities Model

**DOI:** 10.3389/fmed.2021.596873

**Published:** 2021-01-26

**Authors:** Jennifer L. Holcomb, Gretchen H. Walton, Itunu O. Sokale, Gayla M. Ferguson, Vanessa R. Schick, Linda Highfield

**Affiliations:** ^1^Department of Management, Policy and Community Health, School of Public Health, University of Texas Health Science Center at Houston, Houston, TX, United States; ^2^Department of Internal Medicine, McGovern Medical School, University of Texas Health Science Center at Houston, Houston, TX, United States; ^3^Department of Epidemiology, Human Genetics and Environmental Sciences, School of Public Health, University of Texas Health Science Center, Houston, TX, United States

**Keywords:** social determinants of health, patient navigation, accountable health communities model, implementation science, quality improvement

## Abstract

**Introduction:** The Accountable Health Communities (AHC) Model was designed to address the health-related social needs of Centers for Medicare & Medicaid Services beneficiaries. Bridge organizations across the AHC Model have identified lack of technical assistance and peer planning as potential barriers to Model success, particularly around patient navigation. The technical assistance and peer planning literature lacks an organizing, conceptual framework, but implementation science frameworks could serve as useful guides. The Strengthening Peer AHC Navigation (SPAN) research protocol seeks to fill this gap and will apply three implementation science frameworks, Consolidated Framework for Implementation Research, Intervention Mapping, and the Expert Recommendations for Implementing Change compilation, to develop a multi-level quality improvement intervention and evaluate the impact of peer planning on Model outcomes. The aims of the SPAN study are to implement and evaluate a novel multi-level quality improvement intervention to improve AHC implementation and navigation milestones through structured peer planning and to provide successful technical assistance for the AHC Model.

**Methods and Analysis:** The quality improvement intervention is outlined in four Tasks: (1) Assessment – to conduct an assessment of each bridge organization's current implementation, needs, and readiness in AHC Model navigation activities; (2) Planning – to engage in a peer planning approach to build capacity for AHC Model navigation activities; (3) Implementation with technical assistance – Co-creation of a quality improvement protocol for AHC Model navigation activities; and (4) Evaluation – measure the impact of the peer planning and technical assistance approach. Alongside the development and implementation of the quality improvement intervention, this protocol describes a mixed method, convergent parallel study design which will be used to evaluate whether the quality improvement intervention will lead to better outcomes. Tasks will be replicated with five bridge organizations participating in the AHC Model.

**Discussion:** This research protocol provides a framework that can be used to conduct structured peer planning with technical assistance for social needs programs. This study will provide data on both implementation and outcomes which eventually may impact healthcare cost and utilization.

## Introduction

### Accountable Health Communities Model

The recognition of social needs and health-related outcomes, coupled with the Triple Aim developed by the Institute for Healthcare Improvement for improving healthcare has led to national calls for changes in healthcare delivery that consider the social needs of patients. Interventions to address social needs have been tested internationally through frameworks like the Rainbow Model of Integrated Care (RMIC), but a notable gap exists in how to best address social needs in the United States (U.S.) (1, 2). This resulted in innovative integrative strategies across U.S. health systems enhanced by these social prescribing interventions (2–6). The Accountable Health Communities (AHC) Model is a novel approach currently undergoing testing by the Centers for Medicare & Medicaid Services (CMS) Innovation Center's (CMMI) (7). CMMI is focused on developing and testing innovative healthcare payment and service delivery models to improve patient care and healthcare costs for Medicare and Medicaid beneficiaries. Medicare and Medicaid are two separate, government run programs in the U.S. providing health insurance coverage to those who are 65 years of age or older or who have a disability and to those who are low income as determined by each U.S. state, respectively. Individuals can be dual-eligible for both programs. The AHC Model focuses on five social need domains of those covered under Medicare or Medicaid including housing instability, difficulty paying bills, food insecurity, transportation, and interpersonal safety (7). Using a national randomized controlled trial (RCT) with 29 AHC funded sites around the U.S., the trial investigates whether systematically identifying and addressing patient social needs will impact healthcare costs and reduce healthcare utilization (7). Currently, CMMI is testing two tracks of the AHC Model intervention: community service navigation (*Assistance Track*) and community service alignment (*Alignment Track*). In the *Assistance Track*, a bridge organization implements the intervention as a hub and spoke model focused on screening of social needs, referral to community resources, and patient community resource navigation at a clinical delivery site (CDS) [e.g., outpatient clinic or emergency department (ED)] using a RCT. The *Alignment Track* completes the same procedures; however, it is not an RCT and adds a community alignment intervention. The bridge organizations work with community service providers to better align community resources with patient social needs identified through community gap analysis. This track of the Model is based on earlier tests of State Innovation Models and similar models including the Accountable Community for Health (ACH), and Collective Impact approaches (8). Both tracks intentionally allow for flexibility in real-world implementation, are designed for local tailoring, and allow implementers to determine approaches best suited to meeting their communities' needs (8). Patient navigation occurs within both tracks of the Model and represents a focal milestone.

In both tracks, to successfully provide patient navigation, participating bridge organizations must first complete universal screening of community-dwelling Medicare and Medicaid beneficiaries at the CDS, via SMS or text message or over the phone and then provide a referral of “high-risk” beneficiaries to community resources. A “high risk” beneficiary is a beneficiary with a health-related social need who self-reports 2 or more emergency department (ED) visits in the 12-month period prior to seeking care at the CDS. AHC navigator(s) at each bridge organization conduct a detailed personal interview with each patient and assist them in the creation of a patient action plan which is used during follow-up navigation interactions. This process requires navigators to first determine what the essential elements or inputs of a patient action plan include and how to tailor one for their local population (i.e., requires knowledge of patient perspectives about barriers and facilitators to seeking resources) and then how to operationalize it once defined [e.g., software/information technology (IT) or data collection tools].

### Technical Assistance

To better facilitate patient navigation outcomes in the AHC Model, bridge organizations have identified technical assistance (TA) as a priority need. Technical assistance is a blanket term which can encompass a variety of different interventions. To date there is no standard agreement on the definition or essential elements of what comprises a TA intervention (9, 10). A content analysis of working definitions found three over-arching common elements: (1) capacity building (11–13), (2) quality implementation (14), and 3) quality improvement (QI) (15). However, the field lacks consensus on how to effectively implement TA interventions (16). Further, a “good” relationship between the TA provider and TA recipient has been identified as important (17, 18). Katz and Wandersman (16) found relational components including trust, TA recipient's faith or confidence in the TA partner/provider, perceived respect, perceived quality of the TA provider, collaboration, matching strategies with recipient readiness, use of strengths-based TA approaches, use of autonomy-supportive TA approaches, and developing rapport as important to TA success. In a scoping review of TA studies, five phases of TA interventions were also identified: preparation, planning, implementation, evaluation and sustainability (19). As shown in Table 1, five steps in the preparation phase, six steps in the planning phase, five steps in the implementation phase, five steps in the evaluation phase and four steps in the sustainability phase were identified.

**Table 1:** Five phases of technical assistance interventions.

Preparation phase	1) Needs assessment or gap analysis to identify desired changes or improvements 2) Decision-making to establish priorities for TA 3) Visioning of the future state if desired changes were achieved 4) Assessment of staff readiness for and commitment to making desired changes 5) Determination of resources to make the identified changes
Planning phase	1) Setting objectives and goals for program changes 2) Specification of the evidence-based or innovative practices that would be the focus of TA 3) Assessment of whether the proposed intervention practice and TA approach “makes sense” and fits with the existing program mission and goals 4) Development of a logic model, theory of change, or other type of plan for describing the relationships between inputs, practices, and outcomes 5) TA resources that will be made available to facilitate change 6) Roles and responsibilities of staff in learning to use the targeted intervention practices to achieve desired program, organization, or system change
Implementation phase	1) Explicit efforts to establish the credibility of the TA provider and the proposed approach to TA 2) Use of professional development or training to promote staff abilities to use targeted practices 3) Use of coaching or mentoring by the TA provider as part of professional development 4) TA provider consultation in response to staff requests for assistance and guidance 5) TA provider supports and performance feedback in response to progress toward using targeted intervention practices
Evaluation phase	1) Description of process or formative evaluation 2) Outcome or summative evaluation 3) Fidelity of use of the targeted intervention practices 4) Fidelity of use of the TA practices 5) Use of lessons learned to make changes or improvements in practices
Sustainability phase	1) Use of capacity-sustaining activities to sustain or maintain changes 2) Ongoing quality improvement processes 3) Ongoing support from TA provider after intervention practices 4) Follow-up activities between staff and TA provider

*Adapted from (19)*.

#### Peer-to-Peer Approach/Peer Planning

A peer-to-peer approach has been utilized for TA in different implementation settings (20). The approach shares information and tools through the use of interactive discussions such as webinars and conference calls, peer-to-peer consultation, and data sharing (20). It facilitates inter-organizational collaborations and learning from shared experiences of successes, barriers, and developing needs during implementation (21). Hefelfinger et al. (22) evaluated the value of TA and reported that the interpersonal domains including peer-to-peer support and interactions with expert advisors were the most valuable TA resources. Recent research has also shown the importance of co-production in TA interventions. The co-production approach involves each recipient of TA being actively involved in all stages of planning, developing and implementing the TA intervention. This results in programs and assistance that reflect the needs of the organizations and patients (23, 24). Co-production has emerged as an important component of effective and sustainable implementation capacity building in the TA field (25). Co-production includes several important concepts, including co-learning, brokering, facilitation, addressing power differentials, co-design, and tailored support (26).

## Study Conceptual Framework

Implementation science and health promotion intervention frameworks and methods share important characteristics with technical assistance (TA) interventions and may serve as guiding frameworks for the nascent field of TA. Recent implementation science studies have similarly identified the importance of tailoring approaches to local context (27). These studies suggested three steps for successful implementation which include assessing and understanding determinants; identifying change methods (theoretically and empirically based techniques) to influence determinants; and choosing strategies with change methods to address the determinants (28, 29). There are currently limited examples of the application of all three steps in one study in the literature. Our study seeks to fill this gap and will apply three implementation science frameworks to address the three steps above for local tailoring: (1) We will use the Consolidated Framework for Implementation Research (CFIR) to identify determinants, (2) We will use Intervention Mapping (IM) Step 5 (Program Implementation) to identify change methods that are theoretically grounded and (3) We will use the Expert Recommendations for Implementing Change (ERIC) compilation of implementation strategies for strategy selection. CFIR has been used to build implementation knowledge and guide evaluation across multiple kinds of quality improvement (QI) initiatives (30, 31). IM allows for integration of other complementary theories and frameworks to inform the created implementation intervention (32). The ERIC framework was developed to systematically report on implementation strategies and was created through expert consensus (33). The ERIC framework originally consisted of 73 implementation strategies (33). Perry et al. (34) mapped activities from multicomponent interventions to 33 implementation strategies, also adding three new strategies. The 33 ERIC implementation strategies were grouped in four functional groupings: “(1) build health information technology to support data-informed QI, (2) build QI capacity and improve outcomes, (3) enhance clinician and practice member knowledge, and 4) build community connections and patient involvement” (34). The groupings can guide organizations in choosing and combining multiple implementation strategies based on intervention context, needs, and aims (34). These strategies serve as the building blocks of creating a multi-level approach to address identified determinants of implementation for a specific QI intervention (35). The protocol presented in this paper describes the process of designing and evaluating the implementation strategies and existing frameworks and models which were combined to create a new multi-level QI intervention for the AHC Model. We hypothesize that linking three implementation science frameworks in a structured peer planning approach will provide successful technical assistance (TA) for the AHC Model.

### Consolidated Framework for Implementation Research

**Figure 1:**
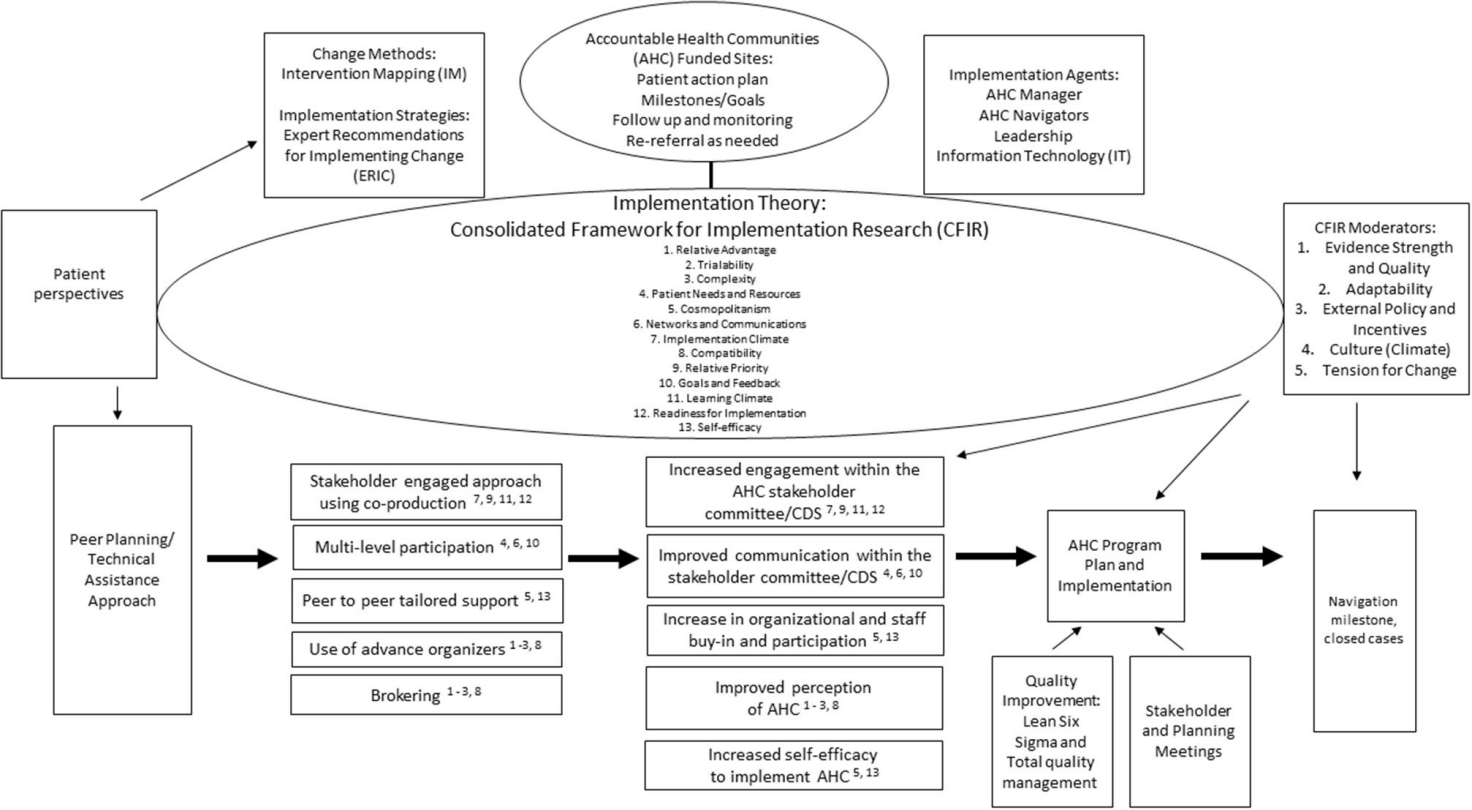
Study conceptual framework.

As shown in Figure 1, CFIR was selected as our overarching framework. The CFIR meta-framework includes five major domains (Intervention Characteristics, Outer Setting, Inner Setting, Characteristics of Individuals and Process of Implementation) across 39 underlying constructs (30, 31). Researchers can prioritize and apply relevant constructs without applying the whole framework to their study (30, 31, 36). The CFIR will be used to identify constructs that we seek to target for behavior change and to identify moderators for evaluation. Intervention Mapping (IM) Step 5 is a structured planning method which focuses on the identification of specific adoption, implementation, and maintenance performance objectives (who had to do what to implement the intervention). It further helps program planners identify determinants of implementation; why clients (AHC decision makers and staff) would adopt, implement, and maintain AHC (28). While actual measures for the CFIR constructs will be tailored, we broadly provide the proposed measurement elements.

Thirteen CFIR constructs were identified in the proposed peer planning and TA approach to support the AHC Model implementation. Three Intervention constructs (e.g., relative advantage, adaptability, trialability) and one Inner Setting construct, trialability, aligned with the use of advance organizers and brokering to improve perceptions of AHC Model. Advance organizers will introduce stakeholders in each bridge organization to the QI protocol development and facilitate peer planning. Previous research has identified this relative advantage, in addition to knowledge and perceptions about the intervention, as tied together (37). To improve perceptions, stakeholders will need to perceive a relative advantage to the proposed intervention vs. an alternative solution (30). Stakeholders' perceptions around adaptability, the degree to which the intervention can be adapted at each bridge organization, and trialability, the ability to pilot the intervention at each bridge organization are important in implementation success (30). There should be a particular focus on consistency in implementation across bridge organizations while also providing adaptability or flexibility to meet local needs (30). Piloting allows each bridge organization to build knowledge about these local needs and the overall intervention to promote successful implementation adaption.

Stakeholder perceptions about implementation adaption success influence the degree of compatibility, the degree of tangible fit between the Accountable Health Communities (AHC) Model and the current norms, values, and existing workflows of each bridge organization. The more stakeholders perceive alignment between the peer planning and TA approach in the AHC Model and their current workflows, the more successful the AHC Model will be. To improve communication between stakeholders through a multi-level approach, one Outer Setting construct (i.e., patient needs and resources) and two Inner Setting constructs (i.e., network and communications, goals and feedback) were identified. The multi-level approach includes input from patients and communication between bridge organization leadership, program champions, and AHC navigators. Organizations that are patient-centered, who understand and meet the needs of patients, are more likely to implement change effectively (30). Organizations who prioritize quality of communication and networks as an organization build a sense of community or “team” (30). Staff receiving clear communication about implementational goals, contributing open feedback to these goals, and receiving peer support to meet these goals can positively influence implementation. Peer-to-peer tailored support increases organizational and staff buy-in, participation, and self-efficacy in AHC Model implementation. Self-efficacy is the individual's belief in their own ability to execute courses of action to achieve implementation goals (30). Staff with high self-efficacy are more likely to embrace and participate in a new intervention (30). A new intervention is also more likely to be embraced with a higher degree of cosmopolitanism, the degree to which an organization is networked with outside organizations (30). A collective of outside networks can increase available organizational resources and stakeholder engagement and improve implementation (30). Stakeholder engagement is also increased by co-production through mutual collaboration from stakeholders involved in AHC Model implementation. Four additional CFIR constructs are aligned with co-production: implementation climate, learning climate, relative priority, and readiness for implementation. The implementation climate is assessed through policies, procedures, and rewards that can be targeted for change (30). The implementation climate sub-constructs include relative priority and learning climate. Stakeholders should share the belief in the importance of the intervention or the relative priority. The learning climate includes the ability for stakeholders and leaders to express fallibility, need and value of others, and ability to try new methods in the intervention (30). These integrated practices enable stakeholder engagement and development to maximize organizational capacity for a new intervention (30). While similar to implementation climate, readiness for change is defined by the more intermediate indicators of an organization's commitment to intervention implementation. A readiness for implementation tool can help to assess organizational and staff capacity and guide a successful implementation (38).

Five CFIR construct measures were identified as possible moderators in the success of the AHC Model implementation and navigation milestones. The Intervention Characteristic constructs of evidence, strength, quality and adaptability have been identified as critical components in engaging stakeholders and ensuring intervention success (30). Stakeholders' perceptions about the strength and quality of the evidence supporting the AHC Model contributes to the credibility of the intervention (30). Evidence can be established through identifying literature, capturing staff and patient experiences, and piloting the intervention (30). The pilot intervention can also help establish adaptability. There should be a particular focus on consistency in implementation across settings while also providing adaptability or flexibility to meet local needs (30). The next two domains are the Inner and Outer Ssettings of the AHC Model implementation. The Inner and Outer Settings of the intervention are not always clear and are dependent on the context of the intervention (30). External policy and incentives can include governmental policy or regulations, external mandates or recommendations, or benchmark reporting from a funder or the public (30). These external forces can create tension between external competing priorities with intervention goals or align with these goals to incentivize implementation. The Outer Setting can influence implementation and can often be mediated through changes in the Inner Setting (30). The two Inner Setting constructs identified as possible moderators are culture and tension for change. Culture is defined as the norms, values, and basic assumptions of an organization (30). In the CFIR, culture has more often been assessed in terms of climate. Climate is localized and tangible compared to the larger construct of culture (30). Tension for change, a sub-construct of climate, is defined as “the degree to which stakeholders perceive the current situation as intolerable or needing change” (30). The desire for change can be driven by current organizational polices in combination with external policies and regulations (39).

## Quality Improvement Approaches

Integrating QI approaches from lean manufacturing into the clinical healthcare space has provided positive and successful results (40). This kind of improvement is deemed “total quality management” and applies both qualitative and quantitative methods to ensure that healthcare processes produce outcomes which achieve desired results (41). *Lean Six Sigma* utilizes a 5-step method to assess if the desired quality of the healthcare outcome is achieved (41). These adapted steps are: define the goals; measure the current process; analyze to verify the relationships and causality of factors; improve the process based on the experimental analysis; and provide control to ensure the variances are corrected (41). This method combines the precision of replicating standards that can be grounded by the scientific method. *Lean Six Sigma* empowers staff with a defined framework for change that can improve work flows and meet identified strategic priorities. Collaboration is the hallmark of this methodology, and because it is process focused it does not interfere with the professional judgment of healthcare practitioners. Management buy-in is extremely important in the maintenance efforts to sustain these changes for the long-term and to empower other site staff and ensure long-term sustainability and success. We will use *Lean Six Sigma* as our QI method for the navigation process. As with traditional *Lean Six Sigma* initiatives, our aim is to improve performance, in our case navigation performance. We differ from the traditional *Lean Six Sigma* practice whereas waste and variation are removed, instead we seek to identify site specific solutions to reach common goals of improved AHC Model implementation and outcomes in the proposed Strengthening Peer AHC Navigation (SPAN) study protocol. The aims of the SPAN study protocol are to implement and evaluate a new multi-level quality improvement (QI) intervention to improve the Accountable Health Communities (AHC) Model implementation and navigation milestones through a structured peer planning and to provide successful technical assistance for the AHC Model.

## Methods and Analysis

### SPAN Protocol

**Figure 2:**
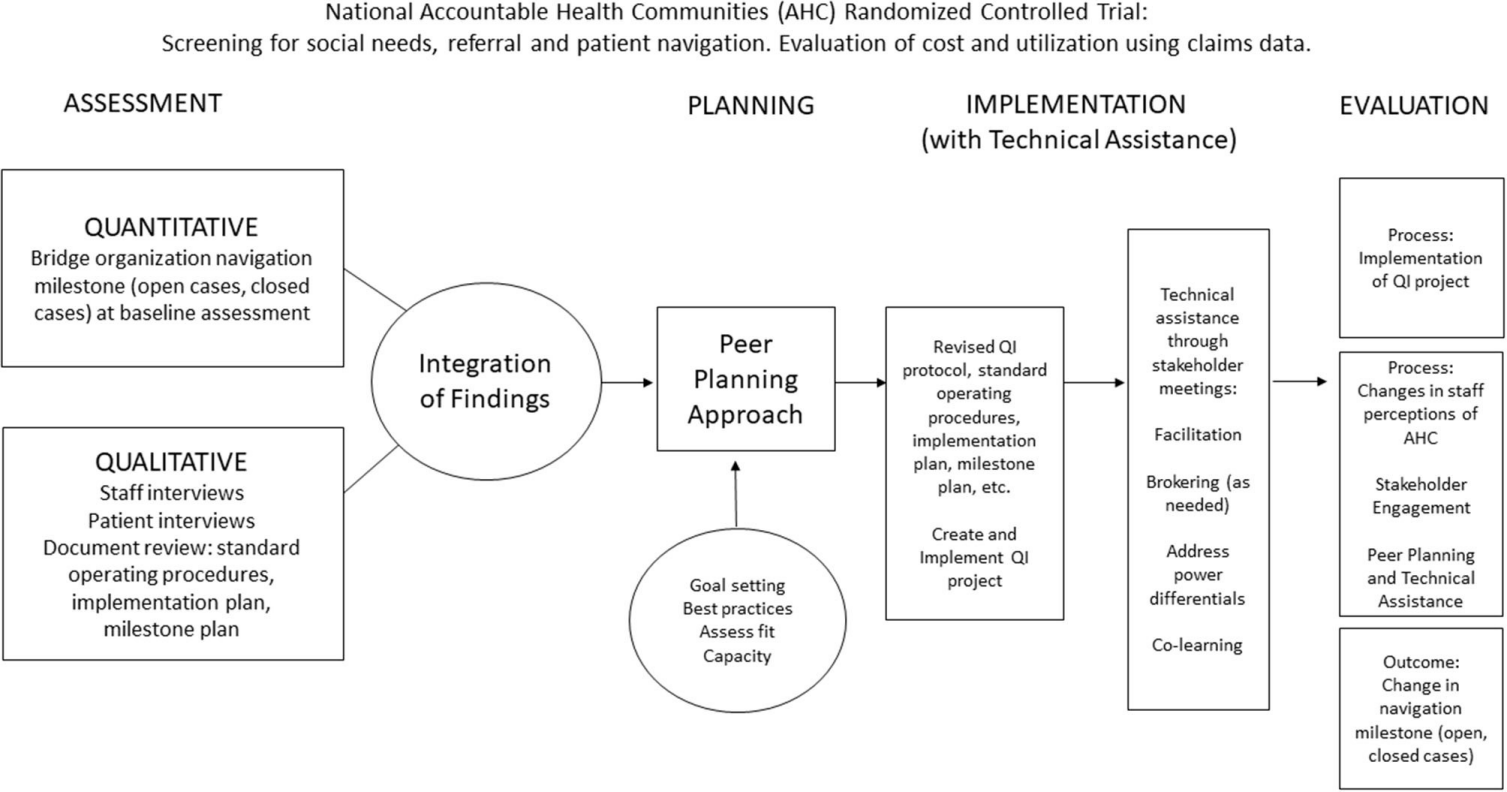
Quality improvement intervention.

The QI intervention in the SPAN protocol is outlined in Figure 2 and includes four distinct Tasks:

- Assessment: Conduct an assessment of each bridge organization's current implementation, needs, and readiness of AHC Model navigation activities
- Planning: Engage in a peer planning approach to build capacity for AHC Model navigation activities
- Implementation w/ technical assistance: Co-creation of a QI protocol for AHC Model navigation activities
- Evaluation: Measure the impact of the peer planning and technical assistance (TA) approach.

Tasks will be replicated with five bridge organizations participating in the national AHC model.

Participating bridge organizations were chosen by an informal assessment of organization implementation needs, and interest in peer technical assistance through conversations between the research team and CMMI program officers. Potential bridge organizations were approached by the research team at the Annual AHC meeting attended by bridge organizations, CMMI and other CMS staff, federal agency partners, and CMS contractors. The research team then worked closely with potential bridge organizations and CMS program officers in follow-up meetings to determine interest and readiness to participate in SPAN. In Task 1, a mixed-method assessment will provide a baseline of the current AHC navigation implementation. Integrated findings from the baseline assessment will guide peer planning to introduce TA resources, to build relational elements and organizational capacity, and to establish priorities and visioning through stakeholder and planning meetings (Task 2). In Task 3, stakeholder meetings will be conducted to develop a quality improvement (QI) protocol and to update current AHC navigation implementation. Alongside the development and implementation of the QI intervention in the SPAN protocol, a mixed method, convergent parallel study will evaluate the impact of the peer planning approach and TA on implementation and navigation outcomes (Task 4). The study design will allow for accurate measurement of selected Consolidated Framework for Implementation Research (CFIR) constructs from Figure 1 while providing a complementary set of data for the overall national AHC Model without harming any desired measures for the larger national AHC evaluation or other outside evaluations. Study results will integrate findings across bridge organizations about AHC activities and navigation outcomes with the evaluation of the multi-level QI intervention and individual QI protocols.

### Task 1 – Assessment of Current Implementation, Needs, and Readiness of AHC Model Navigation Activities

#### Document Review

Through a document review, the current AHC Model activities related to navigation at each bridge organization will be outlined. Documents will include standard operating procedures (SOPs), patient navigation implementation plan, navigation milestone plan, and any other documents relevant to AHC Model implementation. A SOP outlines the steps involved to carry out an implementation specific activity. The purpose of an SOP is to facilitate consistent protocol implementation across CDS and years of the AHC Model implementation as staff members change. SOPs identify the purpose of the activity, owner of an activity, and participants in the activity. The implementation plan walks the reader through the plan for launching the activities required by the AHC Model. This document gives the organizational structure of the AHC Model for each specific bridge organization taking into consideration their CDS. The plan also gives a brief outline of the activities that will be conducted through the course of the AHC Model implementation. The Centers for Medicare & Medicaid Services Innovation Center (CMMI) has set target goals for each bridge organization to reach regarding screening and navigation activities. When the target numbers were changed, bridge organizations were asked to detail how they would alter their activities to meet the new goals. The milestone plan requires that implementation shift to meet the new numbers. The milestone plan will vary by bridge organization as it was up to each bridge organization to decide what course of action, they would take to meet the new numbers.

A keyword analysis will be conducted to develop common codes and themes using NVivo 11 (QSR International) software (42, 43). Relative frequencies and weighted percentages of words will be carried out to determine common codes. Those words with weighted percentages over 1.00%, will be considered for common codes. Using the keyword analysis, similar words will be combined to common codes (e.g., adding managers, teams, etc. to staff). The coding structure will differ by bridge organization and will include any emerging codes. The coding framework will be then used by the research team to code the documents. After the initial coding, the researchers will compare their coding structure and collapse codes into larger key themes. The themes will be considered in terms of their relationships to one another within AHC Model navigation activities and represented through process maps created by the research team.

#### Semi-structured Interviews

The second part of the assessment includes semi-structured interviews with staff and patients involved in AHC Model navigation. Semi-structured interviews with staff will assess perceptions of AHC Model activities, coupled with individual and organizational needs and readiness related to changes in AHC navigation. Semi-structured interviews with patients will assess perceptions and the impact of AHC navigation implementation on their social needs and quality of life. The interviews will expand on the findings from the document review, expanding the understanding of AHC activities and possible moderators to AHC navigation implementation success. Moderators for implementation success identified in Figure 1 will guide the interview questions to address the protocol aims surrounding AHC Model activities and the AHC navigation implementation. Interview guides are available in the Supplementary Material. Interviews will be conducted over videoconference (i.e., WebEx) or phone and verbal consent will be obtained before the start of the interview. We expect to interview at least three staff members and at least five patients from each bridge organization. Staff members will be contacted by email. During routine navigation interactions over the phone, navigation staff will pre-consent 15 patients to be contacted for an interview by the researchers. During the pre-consent, patients will be given the name and contact information of the research team member coordinating the interviews. If consent is obtained, navigation staff will capture patient contact information and a randomly generated number (ID) will be assigned. Once consent from 15 patients has been obtained, navigation staff will provide patient information to the researchers through a password-protected, cloud file approved for the storage of Health Insurance Portability and Accountability Act (HIPAA) information. Patients will be contacted within 2 weeks of pre-consent by phone or email. A standardized pre-consent process and interview recruitment and introduction script will be created. If the patient is not reached after three attempts during the first 2 weeks, attempts to contact that patient will cease.

Interviews will be audio-recorded, transcribed verbatim, and analyzed thematically using NVivo 11 (QSR International). Transcripts will be checked for accuracy through readings and listening to transcripts. Based on the CFIR framework, the research team will create a coding structure for staff interviews and utilize a similar framework but modified for patient interviews. The coding frameworks will then be used by multiple research team members to code each transcript. Codes can be revised, added, and removed throughout the team coding process. The research team members will meet to review individual coding, identify patterns identified with codes, and develop common themes. Interview themes will be used to update the process maps to better reflect navigation activities, as needed. While interviews themes will be identified in each bridge organization assessment, study findings will be described more broadly across bridge organizations.

### Task 2 – Engagement in a Peer Planning Approach to Build Capacity for AHC Model Navigation Activities

The peer planning approach includes stakeholder committee meetings and a two-meeting planning session. The meetings include stakeholders from the research team; bridge organization and clinical delivery site (CDS) staff including organizational leadership, AHC managers, and AHC navigators; and any other key partners in AHC implementation. The stakeholder committee will meet four times over a three-month period with two meetings before the planning session to assess current AHC Model activities and two meetings after the planning session to finalize the QI protocol. Bi-weekly or monthly meetings will be conducted during the implementation of the QI protocol with the committee's discretion. Due to the outbreak of the novel coronavirus in the U.S. in early 2020, each meeting will be conducted over videoconference (i.e., WebEx) in addition to being audio-recorded. Among the research team, there will be at least one facilitator to guide discussion, one co-facilitator to assist with discussion, time management, and note taking, and one note taker to capture a detailed account of each meeting.

In the first stakeholder committee meeting, the research team will review the SPAN protocol and memorandums of understanding (MOUs), roles and responsibilities for committee members will be assigned, a communication plan will be created, and all committee members will agree on the goals for stakeholder meetings. In the second stakeholder meeting, baseline navigation data collection to identify if current navigation milestones are being met by bridge organizations and findings from the assessment (Task 1) will be discussed. The baseline navigation data will be collected from monthly monitoring reports each bridge organization must provide to CMMI. Before the meeting, the research team will present the process maps of the bridge organization's current site activities based on the assessment findings. Bridge organization and CDS stakeholders will review and provide feedback on the process maps during the meeting. During the first planning session meeting, the research team will review and make any additional modifications to the process map(s) created in the stakeholder meeting. Based on the assessment and process mapping, staff will align current implementation strategies with best practices based on the ERIC implementation strategies for multi-component interventions (34). In the second planning session meeting, an IM planning session will be held to focus on areas where QI could be helpful for the AHC site's current implementation approach and focus on prioritization of AHC implementation performance objectives and determinants. The aim of the second planning meeting is to identify between two and four modifiable determinants to be mapped to specific ERIC implementation strategies for QI. The staff will use these strategies to create the QI protocol, revised standard operating procedures (SOPs), implementation plan, navigation milestone plan, and other relevant QI documents at the third and fourth stakeholder meetings.

### Task 3 – Co-creation of a Quality Improvement (QI) Protocol for AHC Model Navigation Activities

Following peer planning, a third stakeholder meeting will be held with each bridge organization to develop a quality improvement (QI) protocol and to update QI documents for AHC Model implementation at the CDS. The QI protocol allows each bridge organization to demonstrate AHC navigation implementation achievement and determine short term impact on milestones. The protocol will build on activities and data collected in Tasks 1 and 2. The fourth stakeholder meeting will focus on key components of a QI protocol and completion of a written QI protocol. As shown in Table 2, the QI protocol will be completed using the Quality Improvement (QI) Bridge Organization Team Charter Template. The template was adapted from QI protocol charters developed by the Agency for Healthcare Research and Quality (AHRQ) Quality Indicators Toolkit (44), Institute for Healthcare Improvement (IHI) (45), and CMS Medicare Quality Assurance and Performance Improvement programs (46). The purpose of the template is to describe the stakeholder committee roles, QI protocol aims, scope and timeline, protocol implementation strategies, and change management strategies (e.g., how to measure QI protocol success). Upon review and approval by each bridge organization, the protocol will be sent to CMMI for review and approval. CMMI leadership and program officers will review the protocol to ensure there is no overlap with existing Model activities or budgetary fund allocation. Upon approval by CMMI, each bridge organization will complete the Quality Improvement Protocol Readiness Assessment Checklist outlined in Table 3 (47). Upon expressing readiness to implement the QI protocol, bridge organizations staff will be assigned to the new protocol, budgetary funds will be released to support implementation by the bridge organization and implementation will begin. A minimum of two additional stakeholder meetings will be held during QI protocol implementation to provide facilitation, support, and ongoing assistance to each bridge organization.

**Table 2:** Quality improvement bridge organization team charter template.

Bridge Organization Name:
QI Protocol Aim:
Bridge Organization QI Protocol Sponsor:
Protocol Background:
QI Protocol Scope:
QI Protocol Implementation Strategies:
QI Protocol Change Management Strategies (take from planning meetings):
Estimated Date for Start and End of QI Protocol:
Team Meeting Frequency during QI Protocol:
Team Members Participating:
Notes:

*Adapted from (44–46)*.

**Table 3:** Quality improvement protocol readiness assessment checklist.

**No**.	**Pre-Implementation Task**	**Yes**	**No**	**If no, why not?**	**Documentation**
**Planning meetings**					
1)	Identified a QI program champion				
2)	Identified implementers				
3)	Completed assessment & planning meetings				
4)	Developed QI protocol based on assessment & planning meetings				
**Stakeholder meetings**					
5)	Actively participated on stakeholder meetings				
6)	Consensus on QI protocol and CMMI reviewed				
7)	Plan for QI check-ins and communication made				
**Staff training**					
8)	Staff training completed if required				
9)	Staff express readiness to start QI protocol				

*Adapted from (47)*.

### Task 4 – Measurement of the Impact of the Peer Planning and Technical Assistance Approach

A mixed-method process and outcome evaluation study will examine the implementation of the overall QI intervention and the QI protocol at each bridge organization as well as the global Accountable Health Communities (AHC) Model navigation implementation. The evaluation will have two layers to assess implementation fidelity, process, and outcomes for both the QI intervention and each QI protocol delivered at each bridge organization.

#### Evaluation of the QI Intervention

The evaluation of the QI intervention will be conducted by evaluators not directly involved in the delivery of the QI intervention. Process evaluation will assess implementation fidelity, and perceptions of those delivering and receiving the QI protocol. Implementation fidelity refers to the extent to which the planned QI protocol and revised standard operating procedures are implemented as planned. For the process evaluation, data will be collected in document review and semi-structured interviews with staff responsible for implementing the QI intervention. The evaluator will review the QI protocol, revised standard operating procedures, and navigation milestone quarterly reports submitted to CMMI and any other relevant QI documents. Coupled with the document review, semi-structured interviews with staff will be conducted to establish the current activities. Staff interviews will also assess the perceptions of the peer planning approach and QI protocol implementation. The staff interviews will be guided by the CFIR constructs used in Task 1 in order to assess staff perceptions and experiences of the QI process.

For the outcome evaluation, the methods and analysis of the document review and semi-structured interviews will reflect methods outlined in Task 1. Semi-structured interviews with the protocol staff will be used to assess progress toward intended outcomes of the QI intervention (e.g., staff satisfaction, increased self-efficacy in QI delivery). A keyword thematic analysis will be conducted to develop common themes to compare planned and current AHC activities. The themes will be considered in terms of their relationships to one another within the QI intervention and AHC navigation implementation. The evaluation team will create a coding structure to develop common themes in order to compare planned and current activities in addition to perceptions of peer planning, AHC navigation implementation, and QI protocol, as appropriate. A modified conceptual framework for implementation fidelity will be used to identify potential moderators (e.g., recruitment, participant responsiveness, comprehensiveness of policy description) to successful implementation (48). The outcomes from the QI protocols (described below) will also be used as metrics of success for the larger QI intervention.

#### Evaluation of the QI Protocols

The evaluation of the QI protocols will be completed by the research team in collaboration with the staff implementing the QI protocol at each bridge organization and the CDS. The staff implementing the QI protocol will evaluate their own protocol in order to provide the staff with the tools and skills needed to conduct and assess future QI protocols (capacity building). Implementation fidelity will be assessed using similar metrics and methods as described for the QI intervention evaluation (see above description). During the planning process for the QI protocol, the research team staff will work with each organization to identify specific outcomes they want to focus on through their QI protocol. The QI protocols identified during this planning phase will be used to inform the data collected for both the process and outcome evaluation. Outcome measures will be based on data generated from weekly data files submitted to CMMI by each bridge organization as required per the Model terms and conditions of award. Weekly files are converted to CMS records and available through the bridge organization monthly monitoring report in the AHC Portal. We anticipate a period of 6 months of intervention data will be available for each site for comparative purposes. Each bridge organization will extract data from their monthly monitoring report for the previous 12 months, which will serve as the baseline data period. Data extracted for each bridge organization will vary per site and protocol based on the staff identified QI protocol and patient interviews but examples include: the unique number of beneficiaries who accepted navigation, the number of core needs resolved from navigation cases, and the number of closed navigation cases with no core needs met. Data for the intervention period (implementation of the QI protocol) will be extracted using the same report and measures. Descriptive statistics [mean, median, range, interquartile range (IQR)] will be calculated for each time period for all measures and compared across time using parametric or non-parametric statistics as appropriate.

## Discussion

It is expected that this research will increase understanding of how a QI intervention through a structured peer planning approach can provide successful TA for improving the AHC Model.

First, despite this study protocol being limited to the U.S. health system, the AHC Model was largely developed from similar social prescription interventions in other countries (2). Due to our focus on the implementation of the Model in the US, our approach may be limited or need adaptation prior to use in other countries with differing health systems. Future adaption of this protocol, particularly with those participating in social prescribing interventions in the U.S., should consider these as potential enhancements in intervention development and QI methods (2). Second, while the bridge organizations participating in the QI intervention reflect a diversity of geographic locations, beneficiary demographics, size, and type (e.g., county governments, hospitals, universities, and health departments), the process for determining bridge organizations used in this study was an informal and subjective process. Future implementers are encouraged to use objective measures with an implementation science basis, to assess organizational needs and site readiness. The inclusion criteria for the QI intervention was also limited to urban or suburban sites as outlined by the funder. Additional research might be needed to explore the implementation of this protocol with rural sites. A strength of the proposed approach in this protocol is our ability to evaluate the impact of TA on the AHC Model. Third, the current study will develop and test a potential conceptual framework for a QI intervention by linking three implementation science frameworks. Using an implementation science framework can help ensure the adoption and sustainability of structured peer planning and TA interventions in both bridge organizations and in other settings (27). The framework will guide assessment and implementation of current AHC Model activities, QI intervention, the peer planning approach, and QI protocols. The TA and peer planning literature lacks an organizing, conceptual framework. There are limited examples of the application of an organizing framework that uses a locally tailored approach to identify evidence-based determinants and develop strategies to address the determinants. The use of the framework will allow for local tailoring in each Task based on each bridge organization's needs while also providing consistency in implementation across bridge organizations. Next, the findings will describe the implementation of a QI intervention through a peer planning approach with TA over WebEx. As there is limited evidence regarding the implementation of peer planning approaches particularly through remote or off-site TA, this protocol provides a way to further test and understand the impact of off-site TA generally (49). Most studies exploring off-site TA have focused on conference calls and online webinars rather than ongoing, personalized interactions with the TA providers. In the QI intervention, off-site TA will be provided through ongoing, interactive video meetings (WebEx) in addition to interactions through email, phone calls, and *ad hoc* meetings with the TA providers and AHC stakeholders. We will embed internal participatory and interactive engagement structures to ensure off-site TA fidelity and success. Lastly a mixed-methods approach will integrate findings from the evaluation of the QI intervention and each QI protocol providing a greater understanding of the global AHC navigation implementation. We will use a variety of data sources such as interviews with the staff responsible for implementing the QI intervention and recipients of the QI intervention (bridge organization and CDS staff), review of documents, and CMMI navigation milestone quarterly reports. This will increase the validity of the evaluation. The evaluation of the peer planning and TA approach at two different layers is an opportunity to identify possible implementation CFIR moderators and evaluate process and outcome navigation measures. To date, evidence from a multi-layer evaluation of the impact of the peer planning and TA approach is limited.

### Anticipated Challenges

While TA research has identified relational and content components, research is limited on TA within the context of relationship building in a peer planning approach. Identified challenges in peer planning include hesitation and lack of enthusiasm from stakeholders, lack of organizational leadership support, and limited capacity (e.g., time and resources) for AHC implementation (50). Strategies for overcoming these barriers include providing a venue (e.g., planning and stakeholder meetings) for stakeholders to discuss organizational successes, failures, and expectations. The meetings were created to engage stakeholders and develop capacity to implement the QI protocol. The roles and expectations of each stakeholder and organizational leader are clarified in these meetings in order to sustain support and engagement. Highlighting AHC successes and patient stories as models for organizational expectations can also assist in engaging organizational leaders. Prior to implementation, the assessment in Task 1 will identify current capacities and which capacities the TA provider should help each bridge organization build. With capacities identified, ongoing stakeholder meetings with TA provide support in implementing and evaluating the QI protocol.

## Conclusion

The study will provide insights into how the implementation and evaluation of a QI intervention may improve implementation and navigation outcomes to better support the AHC Model, and eventually, impact healthcare cost and utilization. The study protocol design has not been applied in the AHC context and provides a framework to conduct a structured peer planning approach in order to provide successful TA. The research will benefit TA researchers and providers in terms of identifying processes in the peer planning approach and to help inform future locally tailored, evidenced-based QI interventions both inside and outside the AHC context.

## Ethics Statement

The studies involving human participants were reviewed and approved by Committee for the Protection of Human Subjects at The University of Texas Health Science Center at Houston (UTHealth). Written informed consent for participation was not required for this study in accordance with the national legislation and the institutional requirements.

## Author Contributions

GW, VS, and LH conceptualized the study. GF and LH developed the study protocol. JH lead pilot testing of the study protocol with assistance from IS, GF, and LH and led manuscript development and prepared the final manuscript. All authors contributed to manuscript writing and revisions. All authors have read and approved the manuscript.

## Conflict of Interest

The authors declare that the research was conducted in the absence of any commercial or financial relationships that could be construed as a potential conflict of interest.
