# Supplementary material for: Developing and Evaluating a Quality Improvement Intervention to Facilitate Patient Navigation in the Accountable Health Communities Model
Source: Front Med (Lausanne). 2021 Jan 26;8:596873. doi: 10.3389/fmed.2021.596873 (PMC7870472; doi:10.3389/fmed.2021.596873)
Supplement: Supplementary file 1 [file Table_1.DOCX]

Supplementary Material

**Consolidated Framework for Implementation Research (CFIR) Staff Interview Guide**

Thank you for taking the time to speak to me today. This is an opportunity for you to share your experiences with the Accountable Health Communities or AHC Model at **[ORGANIZATION NAME].** My name is **[MODERATOR’S NAME]** and I will help guide the discussion today. I will ask some questions and follow-up on points that you make. This discussion should be like sitting down and talking with a friend. There are no right or wrong answers. I am interested in hearing about your experiences with the navigation program and how peer planning of organizations in the Accountable Health Communities Model may improve Model activities, specifically patient navigation. I am interested in learning about the program, what worked, what did not and how it could help your organization.

We will be using a digital tape recorder during the session and will only use first names during the discussion. As mentioned in the consent no identifying information will be included in any program reports. Files will be kept on password protected computers and in locked offices at **[ORGANIZATION NAME].** Do you have any questions before we begin?

I will start the recording now.

**Intervention Characteristics**

I would like to start off by asking about your experience with the Accountable Health Communities or AHC Model at your organization.

**Evidence Strength & Quality**

1. What do you think of the AHC?
   - What was your role? Job Title?

**Trialability and Complexity**

1. Was AHC piloted prior to full-scale implementation in your organization (or CDS)?
   - **[If Yes]** Can you describe your piloting approach and if it was helpful?
   - Do you feel that you can go back to your previous way of practice if AHC is not sustained?

**Complexity**

1. How complicated is AHC?
   - Please consider the following aspects: duration, scope, intricacy and number of steps involved and whether the intervention reflects a clear departure from previous practices.

**Adaptability**

1. What kinds of changes or alterations do you think were needed to be made to AHC so it would work more effectively in your setting?
   - Do you think you will be able to make these changes in your organization? Why or why not?
   - Did you make these or any other changes?

**Relative Advantage**

1. How does AHC compare to other similar existing programs in your setting?
   - What advantages does AHC have compared to existing programs?
   - What disadvantages does AHC have compared to existing programs?

**Readiness for Implementation**

**Available Resources**

1. Do you have sufficient resources to implement and administer AHC?
   - Define when this started for participant.
   - **[If Yes]** What resources are you counting on? Are there any other resources that you received, or would have liked to receive?
   - What resources will be easy to procure?
   - **[If no]** What resources will not be available?

**Access to Knowledge & Information**

1. What kind of training was there for AHC for you? For colleagues?
   - Do you feel it prepared you to carry out the roles and responsibilities expected of you? Can you explain?
   - What are the positive aspects of planned training?
   - What is missing?
   - What kind of continued training is planned or needed?

**Self-efficacy (Characteristics of Individuals)**

1. How confident were you that you could successfully implement AHC?
   - What gave you that level of confidence (or lack of confidence)?
2. How confident do you think your colleagues felt about implementing AHC?
   - What gave them that level of confidence (or lack of confidence)?

Next, I want to talk about your organization, specifically your organization’s current processes and programs, staff, culture, goals, and priorities.

**Inner Setting**

**Learning Climate**

1. Can you describe a recent quality improvement initiative or an implementation of a new program? **[Not AHC]**
   - Can you describe the new initiative/program and the motivation to improve/implement it?
   - Can you tell me the major milestones or key accomplishments along the way?
   - What factors helped make it successful/fail?
   - Who were the key "players"?
   - What was your involvement?
   - Were people happy with the outcome/initiative?
   - Can you tell me about how your supervisor (or other leaders) were involved? Who? Their roles? How they helped/hindered?
2. To what extent do you feel like you can try new things to improve your work processes?
   - Do you feel like you have the time and energy to think about ways to improve things?
   - Did you feel valued/respected by your supervisor for the role you played?
   - What role did your supervisor (or other leaders) play? What actions did they take?

**Networks & Communications**

1. Can you describe your daily interactions with your colleagues?
   - With colleagues in and out of your unit?
   - Can you tell me a story about a time you needed to work with others to solve a problem? Or to implement an intervention in the past or AHC?
2. When you need to get something done or to solve a problem, who are your "go-to" people?
   - Can you describe a recent example?

**Culture**

1. How would you describe the culture of your organization? Of your own setting or unit?
   - Do you feel like the culture of your own unit is different from the overall organization? In what ways?
2. How do you think your organization's culture (general beliefs, values, assumptions that people embrace) affect the implementation of AHC?
   - Can you describe an example that highlights this?

**Compatibility**

1. How well does AHC fit with these values and norms at your organization?
   - Values relating to interacting with individuals served by your organization, e.g. shared-decision making vs. being more directive?
   - Values related to referring to outside vendor-based programs vs. providing services by in-house staff?
2. How well does AHC fit with existing work processes and practices in your setting?
   - What are issues or complications that are arising? Can you give specific examples?
3. Can you describe how AHC is integrated into current processes?
   - How does it interact or conflict with current programs or processes?

**Tension for Change**

1. Do you think there was (or is) a need for AHC at your organization? Is AHC helpful or supportive of existing organization programs or processes? Is it an opportunity for growth?
   - Why or why not?
2. Does AHC help your organization meet the needs of the beneficiaries served by your organization or other organizational goals and objectives?
   - Why or why not?

**Relative Priority**

1. Describe activities or initiatives that (appear to) have highest priority for you (for the organization)?
   - What kind of pressure are you feeling to accomplish this? Where is it coming from? Why?
2. To what extent might AHC take a backseat to other high-priority initiatives going on now?

**Organizational Incentives & Rewards**

1. What kinds of incentives are there to help ensure that the implementation of AHC is successful?
   - What is your motivation for wanting to help ensure the implementation is successful?
2. To what extent do you think your supervisor will consider your role in this implementation in your (next) evaluation? In his/her regard for your work or role?
3. Are there any special recognitions or rewards planned that are related to implementing the intervention?
   - Can you describe them?
   - Will these be targeted to groups/teams/units or individuals?

**Goals & Feedback**

1. To what extent are organizational AHC goals monitored for progress?
   - Can you give an example of monitoring in terms of the type of information, who is informed, and how?
2. Who do you ask if you have questions about AHC or its implementation?
   - How available are these individuals?

**Outer Setting**

**Patient Needs & Resources**

1. To what extent is staff aware of the needs and preferences of the beneficiaries being served by your organization?
   - How "in touch" are staff and leadership with the beneficiaries served by your organization?
2. How well do you think AHC meets the needs of beneficiaries served by your organization?
   - In what ways does AHC meet their needs?
3. Can you share a patient story from your navigation?

**Cosmopolitanism**

1. To what extent do you network with colleagues or people in similar professions/positions outside your setting?
   - What are the venues?
2. What kind of information exchange do you have with others outside your setting, either related to AHC, or more generally about your profession?
   - What professional networking do you engage in? Listservs? Local or national conferences? Trainings?

Now I want to talk you about the planning and implementation of AHC at your organization. We are interested in individuals involved and how AHC has been implemented.

**Current Process**

**Planning**

What have you done to get a plan in place to implement AHC?

What would you still like to do? Is there anything you would like to do that has not already been done?

1. What role has your plan for implementation played during implementation?
   - Is it used to guide implementation of the intervention?
   - Is it used to compare planned with actual progress?
   - Have there been/are there revisions or refinements to the plan?
   - Is the plan shared/reviewed with other stakeholders? How regularly?

**Executing**

1. Has AHC been implemented according to your implementation plan?
   - **[If Yes]** Can you describe this?
   - **[If No]** Why not?
2. Has AHC been implemented according to your SOPs?
   - **[If Yes]** Can you describe this?
   - **[If No]** Why not?

**Engaging & Leadership Engagement (Readiness for Implementation)**

1. What do leaders at your organization think of AHC?
   - Who are these influential individuals?
2. What level of involvement or support has leadership at your organization had so far with AHC?
   - What kind of support have they given you? Can you provide specific examples?
   - To what extent will they influence others' use of the intervention? The success of the implementation?
   - Who are these leaders? How do attitudes of different leaders vary?
   - What types of barriers might they create?

**Opinion Leaders/Formally Appointed Internal Implementation Leaders**

1. Who is leading your implementation of AHC? Who are the key influential individuals to get on board with AHC?
   - How did/will this person come into this role? Appointed? Volunteered? Voluntold?
   - What attributes or qualities does this person have that makes them an effective leader of this implementation? What attributes or qualities does this person lack?
   - Does this person have sufficient authority to do what is necessary to implement the intervention?
2. Who else is involved with leading the implementation?

**Champions**

1. Other than the formal implementation leader, are there people in your organization who are likely to champion (go above and beyond what might be expected) AHC?
   - Were they formally appointed in this position, or was it an informal role?
   - What position do these champions have in your organization?
   - How do you think they will help with implementation? Getting people to use the intervention?
2. Can you describe people's perception of this champion/individual?
   - To what extent do you respect the opinions and actions of the champion?

**Closing**

1. Is there anything else that you would like to discuss or that I missed related to your experiences with the Accountable Health Communities or AHC Model at **[ORGANIZATION NAME].**

**Wrap-up/Open Discussion**

- Have we missed anything?
- Do you have any other comments you would like to add?
- Finally, are there any other concerns or issues we haven’t covered that you would like to discuss?

That concludes our interview today. Thank you again for your time and participation. If you have additional information that you did not get to say or if you have any questions, please feel free to follow up with **[NAME, EMAIL AND/OR PHONE].**

**Patient Interview Guide**

Thank you for taking the time to speak to me today. This is an opportunity for you to share your experiences with the navigation component at [**ORGANIZATION NAME**]. My name is **[MODERATOR’S NAME]** and I will help guide the discussion today. I will ask some questions and follow-up on points that you make. This discussion should be like sitting down and talking with a friend. There are no right or wrong answers. I am interested in hearing about your experiences or the experiences of friends and family with the navigation program at [**ORGANIZATION NAME**]. We are interested in learning about the program, what worked, what did not and how it could help you and others in the community.

We will be using a digital tape recorder during the session and will only use first names during the discussion. As mentioned in the consent no identifying information will be included in any program reports. Files will be kept on password protected computers and in locked offices at [**ORGANIZATION NAME**]. Do you have any questions before we begin?

**Standard Prompts (utilized as necessary)**

- What do you mean by that?
- Can you please tell me a little bit more about that?
- Can you give me an example of that?
- How did that make you feel?
- Why do you believe that is helpful/not helpful?

I will start the recording now.

**Sociodemographic Characteristics**

First, can you first start off with telling me a little about yourself? (Or your family member/family)

1. Please tell me about yourself, your age, etc.

**Healthcare Visit**

Now, we are hoping to learn more about your time at **[ORGANIZATION NAME**]. Try to think back to the most recent time you received care at [**ORGANIZATION NAME**].

1. Can you remember the reason for your visit? Was it an emergency or routine visit? First visit?
2. What services did you receive?
3. How long did you stay at [**organization name**]?
4. During your time there, did you have an experience with the care visit that you would like to share? What made that experience good? Or bad?

**Social Needs Prior To The Visit [CHECK ON THE DOMAINS]**

Now, we would like you to think back before your most recent visit at [**ORGANIZATION NAME**].

HIGHLIGHT DOMAINS MOST APPLICABLE PER SITE CLIENT DESCRIPTION – **FOCUS ON TWO OR THREE:**

- Clinical Care – Access to Care and Quality of Care
- Social and Economic Factors – Education, Employment, Income, Family & Social Support and Community Safety
- Physical Environment – Air & Water Quality and Housing & Transportation

**Clinical Care**

1. Can you tell me about how you decided to schedule or go to (access) [**ORGANIZATION NAME**] for health care?

[Prompts: How long did you wait to see the nurse or doctor or schedule care? Do you have a doctor or other medical provider that you see most often at [**ORGANIZATION NAME**]?]

1. Can you tell me about how you felt about quality of the health care you received at [**ORGANIZATION NAME**]?

[Prompts: How was the health issue addressed? Did you feel your health care needs were addressed?]

**Diet & Exercise – Food Availability**

1. I’d like you to think about access to food. Were there times you felt worried that you would run out of food before you had money to buy more?

[Prompt for healthy food. E.g., “Tell me about your meals. How did you decide what to eat?”]

1. Where do you shop for food for you and your family? Where are you getting your food?

**Paying Bills**

1. I’d like you to think about paying bills, in particular utilities for your current living situation. Have there been times where you haven’t had enough money to pay your bills? Can you tell me about one of those times?

**Community Safety**

1. What about safety outside of your home? Did you ever feel afraid of the people around you?

[Prompts: partner, environment]

**Housing**

Can you tell me about your living situation at the time you were receiving care at [**ORGANIZATION NAME**]? Where was the last place you slept? Were you worried about losing housing?

[Prompts: Did you own or rent the home? How long were you able to stay there? Did you safe in your home?]

1. Can you talk about the quality of your house or apartment? Do you have any issues with mold, appliances not working, pests, or other things?

**Transportation**

1. Finally, thinking about getting to your health care or health related appointments was there a time you could not attend a health appointment due to not having a “ride” or transportation to and from the facility?

[Prompt for transportation. E.G., “Tell me how you usually get to your health appointments. Do you drive? Take public transportation?]

**Organization Navigation Program Process**

1. While at [**organization name**], did anyone talk with you about any of your needs related to [Insert needs e.g., housing, food or other concerns within your household] that we just discussed?

[Prompt for navigation process: Who, When, How/Mode, etc.]

1. How did you feel when the staff at [**ORGANIZATION NAME**] asked you about your non-medical related needs such as food, housing, transportation, etc.? In other words, asked you about things at home or in your community?
2. Did you discuss any of your non-medical needs with the staff and group at [**ORGANIZATION NAME**] that I also asked about today? Is there anything you discussed with them that I did not ask about today?
3. Do you have the same concerns or needs today? How confident do you feel that the concern will be resolved in the near future?
4. ***If did not discuss all concerns.*** You said that you did not discuss the non-medical concerns that we discussed today. Why do you think that is? [Was it the organization, person, process, etc.] Is there anything that they could have done to make it easier for you to discuss or share your needs with them?
5. ***If discussed one or more concerns.*** What happened when you told them about your concern or non-medical need? Did [**ORGANIZATION NAME**] connect you with any services to help address these concerns?

[Prompt for details about the navigation process]

1. When they connected you to services, can you tell me something that [**ORGANIZATION NAME**] did that you found helpful? Can you tell me about something that they could have done better?

**Organizational Culture for Navigation Program**

1. Did you feel that connecting you to non-medical needs was a priority for [**ORGANIZATION NAME**]? Why did you feel that way? Did the staff care about your non-medical needs?
2. Do you feel that the navigation program staff helped address the barriers you face to stay healthy? In what ways did you feel supported or cared for by the navigation program staff?

**Services Provided and Follow-Up**

1. Did [**ORGANIZATION NAME**] help you connect with non-medical services? When they connected you to services, were you able to access them?
2. **IF YOU DID RECEIVE NON-MEDICAL SERVICES.** Did you follow-up with the non-medical services? What types of services did you receive? What was your experience with these services? What happened with the services?
3. Did they ask for you to pay or provide any documents? Ask about reasonableness and barriers.
4. Will continue to seek services there? Did they appropriately address your concern?
5. **IF YOU DID NOT RECEIVE NON-MEDICAL SERVICES.** What do you think would have helped you to receive the non-medical services? In what ways could the navigation to non-medical services change? What would help you and others in the community gain access to the care needs?
6. Can you tell me more about the barriers to accessing services? Transportation, Location, Hours, etc. Also explore non-transportation barriers (e.g., limiting inclusion criteria)
7. Did anyone form [**ORGANIZATION NAME**] follow up with you to see if you received community services from the organizations you were sent to? Did you let [**ORGANIZATION NAME**] know that you received or did not receive the services?
8. Is there anything else you would like for us to know about your non-medical needs? What other needs and services do you feel you need to be healthy?
9. Any suggestions on how [**ORGANIZATION NAME**] can help you to better meet those needs? What else could the staff have done to help you gain access to non-medical needs and connect you to other programs in the community?

**Closing**

Is there anything else that you would like to discuss or that I missed related to your experience receiving medical care and non-medical care from **[ORGANIZATION NAME].**

**Wrap-up/Open Discussion**

- Have we missed anything?
- Do you have any other comments you would like to add?
- Finally, are there any other concerns or issues we haven’t covered that you would like to discuss?

That concludes our interview today. Thank you again for your time and participation. If you have additional information that you did not get to say or if you have any questions, please feel free to follow up with **[NAME, EMAIL AND/OR PHONE].**
